# Gammaretrovirus Infections in Humans in the Past, Present, and Future: Have We Defeated the Pathogen?

**DOI:** 10.3390/pathogens15010104

**Published:** 2026-01-19

**Authors:** Antoinette Cornelia van der Kuyl

**Affiliations:** 1 Department of Medical Microbiology and Infection Prevention, Amsterdam UMC Location University of Amsterdam, Meibergdreef 9, 1105 AZ Amsterdam, The Netherlands; a.c.vanderkuyl@amsterdamumc.nl; Tel.: +31-205-664-853; 2 Amsterdam Institute for Immunology and Infectious Diseases, 1105 AZ Amsterdam, The Netherlands

**Keywords:** gammaretrovirus, class I ERV, HERV, type C retrovirus, cross-species transmission, primate, *Homo sapiens*

## Abstract

Gammaretroviruses are ubiquitous pathogens, often associated with the induction of neoplasia, especially leukemia, lymphoma, and sarcoma, and with a propensity to target the germline. The latter trait has left extensive evidence of their infectious competence in vertebrate genomes, the human genome being no exception. Despite the continuing activity of gammaretroviruses in mammals, including Old World monkeys, apes, and gibbons, humans have apparently evaded novel infections by the virus class for the past 30 million years or so. Nevertheless, from the 1970s onward, cell culture studies repeatedly discovered gammaretroviral components and/or virus replication in human samples. The last novel ‘human’ gammaretrovirus, identified in prostate cancer tissue, culminated in the XMRV frenzy of the 2000s. In the end, that discovery was shown to be due to lab contamination with a murine gammaretrovirus. Contamination is also the likely source of the earlier findings. Complementation between genes of partially defective endogenous proviruses could have been another source of the virions observed. However, the capacity of many gammaretroviruses to replicate in human cell lines, as well as the presence of diverse infectious gammaretroviral species in our animal companions, for instance in mice, cats, pigs, monkeys, chickens, and bats, does not make a transmission to humans an improbable scenario. This review will summarize evidence for, or the lack of, gammaretrovirus infections in humans in the past, present, and near future. Aspects linked to the probabilities of novel gammaretrovirus infections in humans, regarding exposure risk in connection to modern lifestyle, geography, diet, and habitat, together with genetic and immune factors, will also be part of the review, as will be the estimated consequences of such novel infections.

## 1. Introduction

In the family Retroviridae, subfamily Orthoretrovirinae, gammaretroviruses constitute one of the six genera recognized, which are the alpha-, beta-, gamma-, delta-, epsilon-, and lenti- (retro)viruses. Spumaretroviruses belong to the subfamily Spumaretrovirinae. Alpharetroviruses are mainly found in birds, whereas epsilonretroviruses are found in fish [1]. Phylogenetic analysis of reverse-transcriptase (RT) domains and of *env* genes is the usual method to define retrovirus genera [2]. An earlier classification scheme, which has now been abandoned, was based on the budding virion structure observed with electron microscopy (EM). Gammaretrovirus particles, for instance, with a centrally located, dense core and hardly any visible spike proteins, exhibit a “C-type” morphology, like alpha-, delta, and epsilonretroviruses do [1]. Older studies, published around 1960–1980, reporting retrovirus discoveries based on virion type without further sequence analysis, therefore need to be approached with some caution. For instance, the first description of the deltaretrovirus human T-lymphotropic virus (HTLV) was centered on its C-type morphology, and not on the genome structure [3].

At present, only two retroviruses circulate in humans: the deltaretrovirus HTLV and the lentivirus human immunodeficiency virus (HIV). There is evidence that a betaretrovirus, a human variant of mouse mammary tumor virus (h-MMTV), also referred to as human betaretrovirus (HBRV), exists in humans, but no consensus has been reached yet [4,5,6,7,8,9]. The same is true for the presumed circulation of a second deltaretrovirus, bovine leukemia virus (BLV), in humans [9,10].

The current lack of infectious gamma- and, perhaps, betaretroviruses in humans is somewhat surprising. Since these two genera are ubiquitous in vertebrates and have a propensity to target the germline, they abound as heritable endogenous retrovirus (ERV) sequences. An extensive genomic record documents their presence over millions of years of evolution [11,12,13]. The human genome likewise contains many gamma-like ERVs, showing that our ancestors were vulnerable to infections [14]. Germline gamma-related proviruses, or what remains of them, together with epsilon-related viral sequences, are known as Class I ERVs [15]. Class II ERVs are alpha-, beta-, delta- or lenti-like, and class III are spuma-like ERVs. In primate genomes, gamma-like ERVs exceed the number of beta-like ERVs; together they constitute the most abundant ERV group in vertebrates [11]. Recombinant beta–gamma retroviruses also exist, for instance, the Old World monkey (OWM) simian retroviruses (SRVs), and simian endogenous retrovirus (SERV), which have beta-like *gag-pol* genes and a gamma-like *env* [16,17,18]. In primate genomes, lenti-like ERVS are present in some prosimians; spuma-like ERVs are scarce, and delta-like ERVS have not been found [19,20,21,22,23].

Gammaretroviral cross-species transmissions are relatively common, especially between closely related species; class switching is rare [11,24,25]. An example of a class switch is the avian reticuloendotheliosis viruses (REVs), which have recently been transmitted from mammals to birds [26,27]. The ERV burden in a contemporary genome can inform us on the history of retrovirus infections, evolution, and cross-species transmissions in the distant past, since ERVs are demonstrably suitable for conveying this information [28,29,30,31,32,33,34,35]. However, such evidence will never be complete, since absence in a genome is not proof of absence of infection; germline infection and fixation of a trait are equally important for later detection.

To discover current retrovirus infections, diagnostic methods such as serology, Western blotting, PCR, and sequencing can be utilized. Large population surveys for novel retroviruses have not been performed yet, although some early efforts have been published in the past [36,37,38,39,40,41,42,43,44].

Since two retrovirus genera, delta- and lentiviruses, already infect humans, and there is some evidence of human infection with a betaretrovirus, this review will summarize evidence for, or the absence of, another major group of retroviruses, namely the gammaretroviruses. Infection with gammaretroviruses, or the lack thereof, in the past, present, and future will be reviewed in the light of the omnipresence of infectious variants in domestic animals, such as poultry, mice, cats, pigs, and many primate species. The review will also discuss factors influencing the infection risk, including lifestyle, medical interventions, geography, diet, and habitat, together with genetic and immune factors, and the anticipated consequences of such infections.

## 2. Review

### 2.1. Selection of Gammaretroviruses Included in the Review

#### 2.1.1. Selection of ICTV-Listed Gammaretroviruses to Be Included in the Review

The International Committee on Taxonomy of Viruses (ICTV) 2024 list contains 15 exemplar isolates of gammaretroviruses [1,45]. These are REV, Trager duck spleen necrosis virus (SNV), chick syncytial virus, Finkel–Biskis–Jinkins murine sarcoma virus, Harvey murine sarcoma virus, Kirsten murine sarcoma virus, Moloney murine sarcoma virus, murine leukemia virus (MLV), Hardy–Zuckerman feline sarcoma virus, Snyder–Theilen feline sarcoma virus, feline leukemia virus (FeLV), woolly monkey sarcoma virus (WSMV), gibbon ape leukemia virus (GALV), koala retrovirus (KoRV), and porcine type-C oncovirus (PCOV) (Table 1). Hosts of the 15 virus species are birds (3×), mice (5×), cats (3×), primates (2×), pigs (1×), and koalas (1×). Unfortunately, the ICTV list is not very practical when looking for retroviruses that could threaten humans, as it contains seven sarcoma viruses, which are replication-defective and originated through recombination of retroviral sequences with cellular oncogenes [46,47,48]. WMSV likely developed as a defective, oncogene-carrying GALV variant in a pet monkey after co-housing with a GALV-infected gibbon [49,50]. However, the WMSV helper virus is closely related to GALV [51]. The porcine PCOV designation probably encompasses all pig gammaretroviruses, although the sequence provided by ICTV is one of a defective ERV with limited homology to the replication-competent porcine endogenous retroviruses PERV-A, -B, and -C. PERV-A and -B, as well as naturally occurring A/C recombinant viruses, can infect human cells, with some of the A/C recombinants replicating at high titers [52]. Thus, regarding putative infectivity for humans, we are left with eight ICTV-recognized gammaretroviruses: the three avian species, and the mammalian MLV, FeLV, GALV, KoRV, and PCOV/PERV (Table 1).

#### 2.1.2. Selection of ICTV-Listed Betaretroviruses with a Gamma-Type *Env* Gene

The *env* genes of the alpha/gamma/deltaretroviruses (‘gamma-type’ *env*) differ significantly from those of the beta-/lentiretrovirus genus (‘beta-type’ *env*). In contrast to the beta-type Env, gamma-type Env subunits are covalently linked [17]. Furthermore, gamma-type *env* genes encode a highly conserved ‘immunosuppressive domain’ in the transmembrane subunit, and require the viral protease for cleavage of the cytoplasmic tail during virion maturation [18]. Retroviruses with a gamma-type Env use multi-membrane spanning transporter proteins as entry receptors, the majority belonging to the solute carrier (SLC) superfamily of cellular transporters [18]. Recombination involving the *env* gene is common in beta- and gammaretroviruses [17]. Recombinant viruses, with betaretroviral *gag-pol* genes and a gamma-type *env* gene, have been designated type D retroviruses in the past based on virion maturation and morphology. ICTV classifies type D recombinants as betaretrovirus, albeit with the remark that they carry unique Env proteins. The ICTV member species list contains, out of the five betaretrovirus members, three exemplar isolates of such type D viruses: the langur virus Po-1-Lu, Mason–Pfizer monkey virus (MPMV), also known as SRV-3, and NWM squirrel monkey retrovirus (SMRV). Other examples of type D retroviruses are the replication-competent ERVs OWM SERV and feline RD-114.

#### 2.1.3. Replication-Compentent ERVs Not Listed by ICTV

Targeting the germline, putatively through infection of the early embryo, is a hallmark of the beta- and gammaretrovirus replication cycle. The ICTV list contains many exemplar isolates that have integrated counterparts in the host germline. In fact, except for the sarcomaviruses and GALV, all such isolates possess integrated counterparts. Although host defenses suppress expression, many proviruses retain some capacity to replicate and produce infectious virions for a long time after integration. The above list of potential retroviral threats should thus be extended with additional replication-competent ERVs that have shown infectious potential, or for which virions have been detected in cell culture, which are the type D ERVs mentioned in Section 2.1.2., but also the primate ERVs baboon endogenous virus (BaEV), MAC-1 isolated from Macaca arctoides and CPC-1 from the King colobus, Colobus polykomos [53,54,55,56]. Table 1 gives an overview of gammaretroviruses and betaretroviruses with a gamma-type env gene discussed in the review.

### 2.2. Germline Gammaretrovirus Infections in the Human Lineage in the Past

Retroviruses with homology to gammaretroviruses integrated in the vertebrate germline already long before the rise in the order of primates in the late Cretaceous, 70–80 million years ago (mya) [11,57,58]. From then on, gamma- and gamma-like retroviruses, such as HERV-W, HERV-H, HERV-V, HERV-FRD, and HERV-E, continued infecting primates [14,59,60]. HERV-T, entering the primate germline around 43–32 mya, is the only HERV unequivocally clustering with ‘modern’ gammaretroviruses, all others being more distantly related [27,61,62].

After integration, ERVs commonly proliferate for substantial periods of time, but humans did not acquire any new ERV lineage after 30 mya [63]. The only lineage expanding after that time was the betaretrovirus HERV-K, where polymorphic integrations still exist in the human population [63,64,65]. Interestingly, in the genus *Homo*, mainly gammaretroviral-like *env* genes have been domesticated, either as syncytins involved in placentation, HERV-W, HERV-FRD, and HERV-V, or as antiviral proteins, HERV-H (Suppressyn, SUPYN) and HERV-T [62,66,67,68,69,70].

### 2.3. Ancient Gammaretroviruses in OW Primates with No Evidence of Human Infection

Focussing on the OW primates, the African and Asian primate lineage from which humans evolved, it is clear from cell culture, DNA hybridization studies and genome analyses that, especially the Cercopithecinae and Colobinae repeatedly suffered from gammaretrovirus infections during their evolutionary time, for which the proviruses did not end up in the ape/hominoid lineage, and thus presumably did not infect ancestral apes and hominoids [34]. Examples here are, for instance, BaEV, Papio cynocephalus endogenous virus (PcEV), and MAC-1 [54,55,71]. A few of these, including BaEV and MAC-1, can be induced from monkey tissues to produce infectious virions, suggesting that their germline acquisition happened relatively recently [53,72,73,74,75]. Productive infection of a human fibroblast cell line with syncytium formation was seen for BaEV, showing that the absence of BaEV sequences in humans is not due to an infection block at the cellular level [76]. Nowadays, lentiviral vectors pseudotyped with BaEV Env are routinely used to transduce human cells of the myeloid and lymphoid lineages, confirming that BaEV can interact efficiently with human primary cells [77]. However, it must be noted that the BaEV *env* gene was modified in various ways to increase performance. For instance, in one construct, the cytoplasmic tail of the wild-type gene was replaced by that of MLV [77,78]. MAC-1 was originally isolated by coculture of a stump-tailed macaque spleen cell line with the human A549 carcinoma cell line [54]. Cell-free supernatant from the culture was then used to transmit the virus to non-infected A549 cells, showing that MAC-1 can efficiently replicate in human cells [54].

A type D retrovirus, SERV, is also present in OWM, but is again not found in ape or human genomes [16,79]. SERV is closely related to the exogenous type D simian retroviruses, SRVs, which are the causative agents of immunodeficiency and neoplasms in captive macaques [80,81,82]. The African green monkey kidney tissue-derived Vero cell line, widely used for the production of viral vectors and vaccines, can produce SERV and BaEV virions upon chemical induction [75,83,84].

An interesting OWM gammaretrovirus is Pan troglodytes endogenous retrovirus (PtERV1), also known as PtG1a or CERV1, which is, as its name suggests, found in chimpanzee and gorilla genomes, but not in Asian lesser apes and human DNA [29,74,85,86]. The high copy numbers of PtERV1 proviruses in the two African ape genomes are not due to common descent, but arose from independent transmissions [85,87]. Human cell lines naturally expressing the PtERV1 receptor SLC52a2, a riboflavin transporter, are susceptible to infection with PtERV1. Therefore, the absence of the virus in humans has been attributed to either resistance, possibly through the expression of antiviral factors such as APOBEC3 or TRIM5alpha, or to a combination of time and place, with human ancestors not being present in Africa during the time the virus was circulating there [85,88]. Human and chimpanzee TRIM5alpha, but not the gorilla variant, have been shown to be capable of restricting a resurrected PtERV1 core protein [89]. Since the restriction capacity depended on a single amino acid change, it could be that chimpanzee TRIM5alpha mutated or that a variant was strongly selected for after PtERV1 infection [89]. Alternatively, TRIM5alpha activity may not be the only factor protecting against PtERV1 invasion [89].

In summary, although gammaretroviruses have been able to infect the germline from the very beginning of the emergence of mammals, and gammaretrovirus species flourished in OWMs from the late Miocene until the present time, humans have so far avoided becoming infected. At least, germline evidence for infection is lacking. Similarly, the Asian orangutan shows no evidence of having contracted any gammaretrovirus infection after speciation, but African apes have been susceptible to at least one OWM gammaretrovirus, namely PtERV1.

### 2.4. Gammaretrovirus Detection in Human Samples in the Recent Past

Inspired by the finding of viruses in transmissible chicken and mouse malignancies in the first half of the last century, which provides an appealing explanation for cancer development, screening of human samples for oncogenic retroviruses became a favorite topic with many researchers [90,91,92,93]. In the 1960s and 1970s, methods employed were mainly cell culture, inoculation, electron microscopy, and RNA-dependent DNA polymerase assays, which resulted in the detection of type C retroviral particles in or from human malignancies or an indication of retroviral replication. As type C virions are characteristic of both gamma- and deltaretroviruses, hybridization studies or sequence analysis are required for further identification. In addition, using either Mg^2+^ or Mn^2+^ in reverse transcriptase (RT) assays may help to differentiate, as gammaretroviral RT prefers Mn^2+^ over Mg^2+^, with the reverse being true for deltaretroviruses [3]. Type C particles obtained from a cutaneous T-cell lymphoma, of which the RT showed a preference for Mg^2+^, were later indeed shown to belong to a different retroviral lineage, the deltaretroviruses [3]. Studies on the detection of type C virions in human samples without further analysis will therefore not be considered here.

#### 2.4.1. Cell Culture-Associated Gammaretrovirus Detection in the 1970s

McAllister et al. inoculated human rhabdomyosarcoma (RD) cells into kittens and recovered the RD-114 cell line from an ensuing brain tumor [94]. The cell line expressed a gammaretrovirus, which they believed to be of human origin, as it lacked homology to FeLV, or any other ‘well-studied’ type C virus [94]. RD-114 was soon shown to be an inducible endogenous virus of cats, the passaging of the human tissue in kittens being responsible for its ‘human’ infection [95,96,97]. Another type C virus, or possibly two, were isolated from a patient with acute myelogenous leukemia, and showed homology to WMSV and GALV [98,99,100,101]. The virus, named HL23V, was likely the result of a laboratory contamination [102,103,104]. The same was true for a BaEV variant with partial homology to HL23V, isolated from tissues of six leukemic patients, and a distinct type C virus with homology to Rauscher murine leukemia virus, in six patients with either leukemia, Hodgkin’s disease, or multiple myeloma [105,106]. The findings led the authors to speculate that “the results suggest acquisition of at least three types of type-C viral sequences in the human population” [106]. As all the above experiments used either passage of human tissue in animals, or coculture with animal cell lines, and animal material was commonly present and propagated in the labs, contamination from animal sources is the most plausible explanation for the findings. In one of the publications, this option was shortly considered: “although obviously a very remote possibility, it was conceivable that the results described here were due to the selective, accidental contamination of the leukaemic tissues with primate tissues contamination”, since “this laboratory uses DNA from many non-human primates” [105]. But even after detecting 3–8% hybridization of a labeled baboon DNA probe to three out of six human leukemic samples, the authors surprisingly stated that “the human leukaemic DNA samples lacked baboon-specific sequences” [105].

Laboratory contamination of human samples with rabbit DNA led, as late as in 1997, to the presumed existence of a novel human retrovirus, HRV-5 [107,108,109]. Since all the early results were obtained in the pre-PCR era, and were based on virus propagation, virion detection, and DNA hybridization, it is clear that animal gammaretroviruses can readily infect and spread in human cell lines under laboratory conditions.

#### 2.4.2. MSRV Is an Endogenous Retrovirus, HERV-W

When a transmissible retrovirus, multiple sclerosis-associated retrovirus (MSRV), was isolated from multiple sclerosis (MS) patients, it was immediately suspected to be of endogenous origin [110,111,112,113]. Indeed, further analysis showed that MSRV was derived from endogenous retroviral sequences of the HERV-W family of gammaretroviruses [114]. The HERV-W *env* gene product was later identified as syncytin 1, a protein involved in human placental morphogenesis [66]. A systematic review showed that HERV-W sequences are overexpressed in MS, which accounts for the findings in patients [115]. Complementation between fragmented genomes likely accounted for the RT activity measured and the virions observed. Overexpression of HERV sequences under pathological conditions has been a regular source of retroviral ‘confusion’ in the past [116].

#### 2.4.3. The XMRV Case, 2006–2012 

A next gammaretrovirus with homology to murine gammaretroviruses, termed xenotropic murine leukemia virus-related virus (XMRV), was detected in human prostate cancer tissue in 2006 [117]. Subsequent studies associated the virus with myalgic encephalomyelitis/chronic fatigue syndrome (ME/CFS), a finding strongly emphasized by the senior author, which caused quite a stir among the scientific community and the despairing patients [117]. In 2011, after many worldwide follow-up studies, which mostly failed to find the virus in any patient group, XMRV was definitely shown to be a murine contaminant of the original human prostate tumor xenograft that had earlier been passaged in mice [118]. The first reports have been retracted by the editors of the respective journals. In 2005, screening of eight human melanoma cell lines had already shown that half of them were productively infected by MLV [119]. The authors therefore strongly recommended “mandatory testing of melanoma and other human cell lines for contamination with infectious MLV or other animal retroviruses, ……, in order to avoid artificial experimental data”, but clearly their advice was ignored. In 1993, HIV-infected HUT78 cells were found to be co-infected with GALV [120,121]. In 2008, contamination of three human cell lines by MLV or by GALV was reported [122]. In 2009, a 293T cell batch was found to be infected with a synthetic MLV variant [123]. Six further human cell lines were discovered to be contaminated with SMRV after its initial detection in a virus-producing human lymphoblastoid cell line [124,125]. A 2015 screen of 577 human cell lines for MLV found that 19 of them contained MLV sequences, with 17 being productively infected as evidenced by PCR analysis, sequencing, and reverse transcriptase activity [126]. Passaging of human tumor cells in immunodeficient mice and the use of murine feeder cells in cell cultures were proposed as the most likely explanations for the findings [119,126]. Once a cell line is infected with MLV, further contamination of other cell lines exacerbates the problem. Sometimes a subclone, but not the original cell line, was found to be MLV-positive, as was the case for the HeLa subclone BT-B [126]. In the same study, blood samples from 30 healthy volunteers were all negative for MLV [126]. Thus, the widespread laboratory contamination of human cell lines with mouse-derived gammaretroviruses again underlines the ease with which human-derived cells can be infected, and demonstrates their ability to support the subsequent replication steps.

A summary of human “rumor” viruses up till 2008, many of them being HERVs, is provided by Voisset et al. [116]. False human gamma- and type D retrovirus detections discussed in the review are listed in Table 2.

### 2.5. Current Infectious Gammaretrovirus Species

#### 2.5.1. Infectious Avian Gammaretroviruses

The ICTV-listed avian gammaretroviruses, three strains of REV, constituted an interclass transmission of a mammalian gammaretrovirus in the recent past [26,27,127,128]. Wildtype REV is not infectious for human cell lines [129,130]. However, an earlier study reported that SNV can infect HeLa cells with the establishment of an integrated provirus, but that a posttranscriptional block prevents viral replication [131]. REV infection in poultry can be diminished by testing and selection, but flocks can still become infected through infection, or vaccination with fowlpox virus or Marek’s disease virus (a herpesvirus) vaccines, due to REV’s ability to integrate into the genome of large DNA viruses [130,132,133,134]. Fortunately for humans, avipoxviruses, of which fowlpox is the prototype, have a very limited host range, infecting only avian species, in contrast to other poxviruses [130].

#### 2.5.2. Infectious Mammalian Gammaretroviruses

Research summarized in Section 2.4.1 on contaminating mammalian gammaretroviruses in human cell lines showed that many non-avian members ofs the genus, especially the MLV-like viruses, are capable of infecting humans at the cellular level [135]. Certain porcine PERV variants also have the ability to do so, with integration of a provirus, although they have not been observed to replicate or spread in humans after xenotransplantation of pig tissue [136,137,138,139]. GALV replicates efficiently in a human T-cell line, but very poorly in human primary T cells [122,140]. KoRV type A, KoRV-A, replicates very well in the human HEK293T cell line, while lentiviral vectors pseudotyped with the wt KoRV-A *env* gene efficiently transduce human primary monocytes as well as B- and NK-cells [141,142,143]. An Australian bat virus related to KoRV and GALV, Hervey pteropid gammaretrovirus (HPG), is also able to infect human (though not mouse) cells [144]. Since GALV and KoRV are closely related, it has been suggested that they originate from a common source, such as a rodent or possibly a bat species [31,49,50,145,146,147]. GALV was first reported in captive gibbons from research colonies, while it could not be isolated from 76 gibbons kept in North American zoos, nor in 23 gibbons from seven species residing in the European Union [148,149]. Still, anti-GALV capsid p30 antibody prevalence was 28% in the American zoo gibbons, whereby *Nomascus* gibbons showed a higher prevalence than *Hylobates* and *Symphalangus* gibbons [148]. It was not investigated whether the seroprevalence could be due to antibody cross-reactivity to, possibly, ERV epitopes [149]. Earlier, Kawakami et al. reported shedding of an infectious C-type virus by four healthy *Hylobates* gibbons kept as pets or in zoos, and by two laboratory animals, though it is not clear whether or not the virus was GALV [150]. GALV can be transmitted between animals, but there is no evidence for seroconversion or virus replication in human gibbon handlers, nor were any of the 338 pre-treatment samples in a clinical trial with GALV-*env*-carrying retroviral vectors positive for such sequences [151,152]. Conversely, it has been speculated that infected humans were the source of GALV in gibbons [153]. Surprisingly, GALV infection in gibbons was not reported beyond the 1970s, strengthening the hypothesis that iatrogenic transmission in US research facilities and not natural infection in the wild was the source of the reported cases [149]. Another monkey virus, SRV4, isolated from Japanese macaques with an infectious hemorrhagic disease, has been reported to replicate in human cell lines and in humanized mice [154].

A post-transcriptional block was observed for FeLV type B replication in human PBMC, although most human cancer-derived cell lines, as well as human non-transformed keratinocytes and lung fibroblasts, were fully permissive [155]. FeLV type A has a low affinity for the human variant of its receptor, thiamine transport protein 1 (THTR1), and is thus an unlikely candidate for a zoonotic transmission [156]. Natural isolates of FeLV commonly consist of mixtures of FeLV-A and -B and can be shed in cat saliva and other bodily fluids [155]. A survey of veterinary conference attendees for evidence of zoonotic infection by feline retroviruses detected no anti-FeLV antibodies nor viral DNA in the highly cat-exposed participants [157]. However, virus transmission from local domestic cats to other felines, namely to Florida panthers, Iberian lynx, and North American pumas, has been recorded [158,159,160].

#### 2.5.3. Replication-Competent ERVs and the Type D Retroviruses

Both endogenous SERV, as well as exogenous SRVs, have the capacity to generate virions, while SRVs can spread within primate colonies [75,80,81,82,83]. It can be speculated that, given their high sequence homology to SERV, exogenous SRVs, including MPMV/SRV3 and Po-1-Lu, at least partly derive from monkey ERVs [82,161].

Simian D-type viruses and the feline ERV RD-114 do replicate in human cell lines, with the induction of RT activity and syncytium formation [76,94,162,163]. The same is true for other replication-competent ERVs, such as BaEV, MAC-1, and CPC-1 [53,54,164]. However, being able to replicate in a cell line is not a reliable predictor of the ability to replicate in primary cells or to productively infect an organism. Evidence for a simian D-type retrovirus infection, with virus isolation, serum reactivity, and PCR amplification of viral DNA, was found in Burkitt’s-type B-cell lymphoma biopsies obtained from a patient with AIDS [165]. As the patient had no exposure to monkeys, and an autopsy was denied, the authors could not completely rule out contamination since type D viruses were also discovered as cell line contaminants [125,165,166,167]. Lerche et al. later reported serological evidence for a type D virus infection in two healthy individuals occupationally exposed to monkeys [168]. However, the virus could not be isolated, PCR-amplified, or transmitted, suggestive of very limited or non-existent replication.

Since gammaretroviruses are frequent laboratory contaminants but have not yet been isolated from humans, it is likely that they need to overcome significant obstacles to accomplish successful cross-species transmission. That observation corresponds with the lack of any gammaretrovirus on the 2024 WHO priority pathogens list, where HIV (Lentivirus humimdef1) is the only retrovirus assigned a Public Health Emergencies of International Concern medium risk level for pandemic potential [169]. Still, the relatively common cross-species transmissions of gammaretroviruses observed, the ease of human cell culture infections, and the many animal species carrying infectious variants that surround us should keep us alert.

### 2.6. Genetic and Immune Factors Associated with Gammaretrovirus Resistance in Humans

Some time ago, it was seen that the so-called animal RNA tumor viruses were effectively lysed by human serum through complement activation [170]. No such effect was found for HTLV and HIV [171,172]. Later, it was shown that inactivation through the complement pathway depends both on the producer cell line, thus the host species, and the viral Env protein, and is therefore an unpredictable mechanism to prevent virus infections [173]. Over the evolutionary timespan, humans have lost the ability to synthesize various carbohydrate antigens, resulting in a loss of certain membrane-associated glycans, such as the complex alpha-gal epitope and N-glycolyl neuraminic acid (Neu-5Gc) [174]. The alpha-gal epitope was lost early in the evolution of the OW primate lineage, but is still present as a major carbohydrate antigen in non-primate mammals, prosimians, and NWM [174]. Naturally occurring human anti-alpha-galactosyl antibodies recruit complement factors to inactivate retroviruses expressing the target carbohydrate on their envelope, offering a defense against zoonotic infections [174,175]. However, depleting anti-alpha-gal antibodies did not enhance the risk of PERV infection in experimental pig-to-baboon organ transplantations [176].

Should an incoming virus have survived the first innate immune barriers, receptor incompatibility would be the next hurdle. Retroviruses with a gamma-type Env use a member of the SLC superfamily as a cellular receptor [18]. Viruses of the so-called RDR superinfection interference group, comprising, amongst others, BaEV, RD-114, the primate D-type viruses, and the avian gammaretroviruses, all use the neutral amino acid transporters SLC1a4/ASCT1 and/or SLCa5/ASCT2 for entry [18,32]. Human syncytin 1, a repurposed HERV-W Env, also binds to SLC1a4/5 [177]. SLC20a1/PiT1 is used by, e.g., KoRV-A, FeLV-B, and GALV [18]. Competition for entry by another Env using the same receptor can lead to superinfection resistance, whereby an incoming virus cannot enter a cell as its receptor is already occupied or downregulated by another Env [178]. ERV Envs can be domesticated to become part of the innate immune system. Exapted HERV-T Env downregulates its receptor monocarboxylate transporter-1 (MCT-1/SLC16a1), and can likely restrict other MCT-1-using viruses [62]. Human suppressyn acts against SLC1a5/ASCT2-using retroviruses at the maternal–fetal interface, while HERV-W Env is capable of restricting avian SNV infectivity through SLC1a4 [69,70,179]. SLC1a4/5 proteins are well conserved among vertebrates, although chickens have lost the SLC1a5 gene [180]. Interferon-stimulated genes (ISGs) are genes activated after interferon release following virus infection [181,182]. Such restriction factors are, for example, the APOBEC3 family, TRIM5alpha, tetherin, SAMHD1, the HUSH complex, ZAP, and SERINC5; they inhibit the viral replication cycle at specific points [183,184,185].

Thus, viral proteins themselves are recruited by the innate immune system to combat further virus infections, showing that host defenses are surprisingly adaptable. Still, the immune system can never be 100% efficient. In immunocompetent individuals, the majority of infections would probably be prevented, but this may be different in those with a genetic flaw or an acquired immunodeficiency. However, the combined action of multiple antiviral mechanisms may explain why, despite regular exposure to animal gammaretroviruses, humans appear to be quite resistant to infection.

### 2.7. Exposure Risk and Gammaretrovirus Infections in Humans

#### 2.7.1. Natural Exposure to Gammaretroviruses

The most effective way of not becoming infected is, of course, not being around when and where a virus is circulating. It has been speculated that humans did just that when PtERV1 was spreading to ancestral chimpanzees and gorillas in Africa, some 3–4 mya [85]. Possibly, ancient hominids were in Eurasia at that time, or alternatively, they lived in a different habitat, such as a savannah environment instead of a tropical forest [85]. Another explanation, although less appealing to the imagination, would be incomplete lineage sorting, with a prehistoric loss of provirus-carrying chromosomes due to the relatively small effective population size of human ancestors [85].

It has been shown that shared habitats are, and have been, important for natural cross-species transmission, although, for instance, bats may carry retroviruses over long distances within their territory [28,30,49,186,187,188]. Retrovirus particles are unstable in the environment and therefore need close contact between individuals for transmission, that is, a direct exchange of body fluids such as saliva, breast milk, or blood. Thus, exposure to a virus-carrying species, both in geography and in ecology, as well as through behavior and diet, e.g., hunting for meat, with the danger of being scratched or bitten, is important for a zoonotic transfer [189]. A systematic analysis suggested primates to be the most likely source of future human retrovirus infections, both through bushmeat hunting and via laboratories [190]. Indeed, all retroviruses found to date in humans, namely human immunodeficiency virus (HIV), human T-cell leukemia virus (HTLV), and foamy viruses, are derived from non-human primates. Two simian immunodeficiency virus (SIV) species, from African apes and sooty mangabeys, respectively, have been transmitted, likely through hunting and bushmeat preparation, resulting in human-adapted lentivirus genera HIV-1 and HIV-2, respectively [191,192,193,194]. Four transmissions of ape SIV in the last century or so gave rise to the current HIV-1 group M, N, O, and P viruses [191,192,195]. The deltaretrovirus species HTLV 1-3 have been transmitted from OWM starting around 30,000 years ago [196]. More recent cross-species transmissions are rare, but do occur [197,198,199,200]. Simian foamy virus (SFV) infections are prevalent in African hunters and in laboratory personnel, and are mainly associated with biting accidents [201,202,203]. Surprisingly, despite the high prevalence of spumaretroviruses in primate species worldwide, including the three ape species, there is no genuine human foamy virus variant [204]. Gammaretroviruses carried by extant primates are mainly found in OWM, such as the exogenous viruses SRV and GALV, and a variety of replication-competent ERVs. Their primate hosts can be encountered in the wild in Africa and Asia, but are also sold as pets, kept in zoos, maintained in temples, and held in primate research centers, facilitating ample human contact opportunities. Non-primate animal species could be another reservoir for zoonotic viruses, with rodents and bats being prime suspects. In addition, there is intensive hunting in the US of mule deer, which carry a recently integrated cervid endogenous gammaretrovirus, CrERV, that can form infectious, xenotropic virions upon coculture with human HEK 293T cells [205]. However, domestic animals are probably a much bigger source of viruses due to their large numbers and their proximity to humans. The combined biomass of humans and domestic mammals increased around fivefold since 1850, while the biomass of wild mammals decreased by 50%, so that the latter now comprises only around 10% of the total mammalian biomass on earth [206]. Exposure risks will be further discussed in the next sections.

#### 2.7.2. Man-Made Exposure to Gammaretroviruses

##### Exposure to Domesticated Animals

In Eurasia, the domestication of livestock, beginning around 6500 years ago, enabled the transmission of zoonotic pathogens that were not found in humans before that time [207]. The ongoing surge of the human population, accompanied by growing numbers of livestock and pet animals, as well as human-enforced declines in the natural habitats of wild animals, makes the human species progressively vulnerable to pathogens carried by animals. Furthermore, humans today are also exposed to semi-domesticated animals, that is, members of wild species kept in captivity and handled by human caretakers, especially when they are young, sick, or dead. KORV, for which some germline integrations date to 22,200–49,900 ya, shows that infectious counterparts of intact ERVs can continue to exist for quite some time [208]. In an analysis of vertebrate genomes, Wang and Han showed that 73 species associated with humans carry genome-invading ERVs, some clustering with gamma-ERVs, suggesting that those could be a source of novel retroviruses [13]. In addition, dogs have been shown to express a young, polymorphic gamma-ERV, CfERV-Fc1(a) [209]. Interestingly, (in)breeding and hybridization associated with domestication have been reported to increase the number of ERV loci in both pigs and mice, together with the occasional generation of recombinant ERVs with novel characteristics [13,135,210,211]. In contrast, CfERV-Fc1(a) already showed amplification in the Old World wolf lineage before the domestication of dogs [212]. Thus, people have been intensifying their contact with animals on a global and industrial scale for quite some time. But, this practice has not yet resulted in a detectable gammaretrovirus infection. However, transmission of the betaretrovirus MMTV from mice to humans at least 4500 years ago has possibly resulted in a human-adapted variant implicated in certain types of human breast cancer and in biliary cirrhosis [5,8,9]. If the finding holds true, the wild mice attracted to the grain stores of early farmers could have been the source of the virus. Another presumed retrovirus transmission related to domestication would be BLV from cattle, whereby positive PCR results were related to the consumption of dairy products such as milk and beef. BLV can infect human cells and has been associated with breast cancer, just like human MMTV [213] (see also [214,215]). Multiple BLV haplotypes were detected in humans, but human-to-human transmission has not been observed [216].

##### Exposure Through Cell Culture Products

Modern biomedical practices and clinical inventions would be another way of confronting the human population with a gammaretrovirus. As summarized in Section 2.4, various gammaretroviruses have been found to contaminate human cell lines in laboratory settings. In a worst-case scenario, such a virus could continue to adapt and maybe escape from the lab. However, cell culture contaminations have been reported for decades, with, again, no discernible human gammaretrovirus infection. Inoculation of cell culture products into a large number of humans, as is performed with live-attenuated vaccines, could enhance the chances of a productive infection. RD-114 virions have been detected in live attenuated vaccines for dogs made in a cat-derived cell line [217]. The infectious RD-114 present in the Crandell–Rees feline kidney cell line used here originated from recombination between different proviruses [218]. Although RD-114 can use canine SLC1a4/5 for entry, no productive infection was detected in dogs subcutaneously inoculated with a virus stock [219,220]. From some human live-attenuated vaccines, especially the Vero cell line-produced Rotateq and Rotarix, both oral anti-rotavirus vaccines, SRV DNA could be PCR-amplified. As DNase treatment eliminated the signal, contaminating African green monkey DNA, but not viral RNA, had probably been present in the vaccine batches [221]. Subsequently, it was shown that ‘SRV’ sequences from the Vero cell line were in fact endogenous SERV DNA [75]. SERVagm–Vero did not replicate in human A-204 and Raji cell lines [75]. A study in Japan, analyzing five Vero-based vaccines, plus 783 clinical samples obtained from patients with various diseases and 1000 samples from healthy controls, found no evidence of SERV infection, except that eight healthy controls > 30 years showed seropositivity for a SERV Pol antigen, but not for Gag or Env [222]. The vaccines analyzed, all produced in Vero cells since 1980, were three inactivated and two oral live-attenuated vaccines [222]. Live-attenuated mumps, measles, and rubella and yellow fever vaccines manufactured in chicken embryos or in a chicken embryonic fibroblast cell line were shown to be contaminated with the alpharetrovirus avian leukosis virus, but human infection in recipients was not observed [36,221,223,224,225]. Of course, biotechnology products made in mammalian cell lines, such as monoclonal antibodies and vaccines, are nowadays routinely screened for contaminating viruses, but no procedure will be 100% reliable [226,227].

##### Exposure Through Animal Tissue and Organ Transplantation

Another man-made medical intervention, transplantation of animal tissue or complete organs in humans, which requires lifetime immune suppression, has sofar not resulted in BaEV or PERV infection in recipients of baboon bone marrow, baboon liver, or various pig tissues [228,229,230]. The first xenotransplantation of a kidney originating from a pig carrying 69 genomic edits, including the inactivation of 59 PERV loci, has been reported [231,232]. Other companies aim to reduce the PERV content in pigs destined for xenotransplantation procedures by selective breeding [233,234,235]. A clinical trial using kidneys from pigs with 10 genomic edits, none of them PERV elements, has started recruiting in August 2025 [236]. As neutralizing antibodies against PERV can be elicited in rats, the development of an anti-PERV vaccine to protect recipients of pig organs has been suggested as an alternative approach [237]. Guidelines for xenotransplantation safety are being updated regularly, and include PERV screening strategies for both donor pigs and human recipients [238,239,240,241].

##### Exposure Through the Clinical Use of Retroviral Vectors

Introducing a gene of interest into humans has been performed using MLV-based gammaretroviral vectors [242]. Since safety issues arose, the use of so-called self-inactivating (SIN) vectors effectively reduces the chances of creating a gammaretrovirus in a patient [242,243]. Here, three plasmids, one coding for *gag-pol*, a second for *env*, and a third carrying the gene of interest flanked by partially defective LTRs, are used to produce viral particles in vitro. Another way of protecting laboratory personnel against MLV when working with lentiviral pseudotyping systems is the use of an ecotropic, thus mouse-only, MLV *env* gene, together with transient expression of its receptor, SLC7a1, in the human producer cell line [244]. In addition, alternative delivery methods for nucleic acids, which eliminate the need for retroviral vectors, have now also been developed [245]. However, not everyone has abandoned the clinical use of replicating viral vectors. A worrisome development is the use of non-human viruses as oncolytic or, despite the above-mentioned safety concerns, as gene-delivery agents in cancer treatment. Koppers, Lalic, and Hoeben estimated the ‘relative environmental risk’ of these replicating retroviral vectors (RRVs) as ‘high’ [246]. RRVs, based on either MLV or GALV equipped with a transgene cassette, have been tried in mouse models first; MLV RRVs subsequently entered clinical trials [247,248,249]. One study analyzed patient samples from a trial on glioblastoma treatment with an MLV RRV, Toca 511, expressing a modified yeast cytosine deaminase to convert the prodrug 5-fluorocytosine into cytotoxic 5-fluorouracil [250]. In patients with detectable viremia, the development of neutralizing antibodies coincided with viral sequences disappearing from the blood [250]. Patient-derived viral sequences suggested APOBEC3-activity during reverse transcription [250]. In 13 patient samples sequenced, MLV did not revert to wildtype, in line with the insert having been reported to be quite stable when directly cloned after the stop codon of the *env* gene [249,250]. A 2023 study reported no evidence of the presence of replication-competent retrovirus (RCR) in 338 pre-treatment and 1595 post-treatment peripheral blood samples obtained from 60 clinical trials [152]. The authors focused here on RCR during the production process of retroviral vectors to be used in clinical trials, exposing the participants to a replicating virus, not so much on the generation of RCR in treated individuals. In the 1990s, RCR MLV recombinants originating from a producer cell line were found to be the cause of lymphoma in three rhesus macaques receiving experimentally transduced bone marrow cells [251,252,253]. The majority of the studies analyzed in 2023 had used vectors with intact long terminal repeats (LTRs), pseudotyped with a GALV Env [152]. In line with the above results, Farley et al. recommend unifying and reducing testing of third-generation retroviral vector systems, except that they “note that there are conditionally replicating RVs (crRVs) being used for cancer therapy, where generation of RCV (replication-competent virus) risk must be considered in a different light” [254].

**Figure 1:**
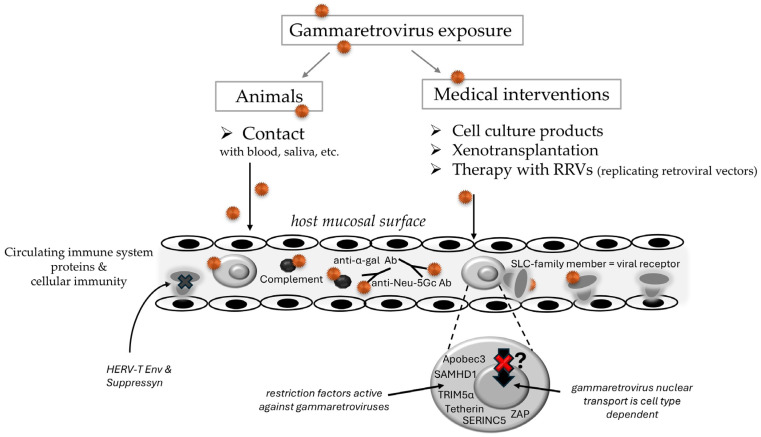
Potential sources of and defenses against a future human gammaretrovirus infection. To enter the human host from an animal source, direct entry of viruses, indicated by orange dots, contained in blood or saliva into the bloodstream through wounds induced by biting or scratching would be the most likely route. All gammaretroviruses known use an SLC-family member for cell entry [18]. Cells and molecules of the innate immunity restrict virus infections, often in a virus-specific way [185]. Some examples are depicted, for instance, natural anti-carbohydrate antibodies that target enveloped virions coming from non-human (anti-Neu-5GC Abs) or non-primate hosts (anti-α-gal Abs) [174]. Exapted HERV Envs can interfere with entry through receptor downregulation, such as HERV-T Env downregulating MCT-1/SLC16a1, and Suppressyn interfering with SLC1a5/ASCT2-using retroviruses in the developing placenta and early preimplantation embryo [62,70]. Examples of cell-associated restriction factors, like Apobec3 and TRIM5α, are also shown [185]. For simple retroviruses, viral core transport to the nucleus is uncommon and cell-type dependent [259,260]. Thus, gammaretrovirus replication occurs mainly in dividing cells, when cell cycle passaging leads to breakdown of the nuclear envelope.

A list of gammaretrovirus threats to humans, as well as their most likely transmission sources with recommendations to decrease the likelihood of transmission, is provided in Table 3. It must be remarked that almost all listed gammaretroviruses exist in an endogenized form in their natural hosts, whereby the probability of provirus activation decreases with time since endogenization. For instance, SERV activation in Vero cells requires chemical induction, while PERV needs mitogenic stimulation to be released from pig primary cells [75,255]. Possible virus sources, as well as factors counteracting or stimulating a human gammaretrovirus infection, are shown in Figure 1.

In summary, animal domestication combined with a human-induced decline in the habitat of wild animals, as well as current healthcare inventions, such as vaccination, xenotransplantation, and retroviral vector therapy, facilitate exposure of the human population to animal gammaretroviruses. But, to date, no replication-competent virus variant, let alone a circulating virus, is known to have emerged from these activities.

### 2.8. Consequences of Novel Human Gammaretrovirus Infections

Assuming a productive novel gammaretrovirus infection, consequences for humankind can be envisaged on both the short term, related to morbidity and mortality from the infection, and on the long term, by influencing evolution through provirus integration in the germline. In order to have a significant effect, the novel virus should be transmissible and not be limited to a single individual, which may be the case in clinical trial participants or in immunosuppressed individuals. As retroviruses spread through intimate contact, e.g., between sexual partners, from mother to child in utero, or through breastfeeding, sufficient opportunities to do so would be essential. Between humans, transmission through biting and scratching is less likely. However, blood transfusion, surgical procedures, or the use of dirty needles could lead to healthcare-associated transmission. In addition, unlike what is known for HIV and HTLV, gibbons and cats can also shed infectious GALV and FeLV, respectively, in urine and feces, while infectious FeLV is consistently detectable in the saliva of viremic cats [150,261,262,263,264]. GALV production was found to be very high in the post-mortem material from the oral cavity of a gibbon with lymphocytic leukemia, likewise implicating the possibility of oral transmission [265].

Regarding pathology, the frequent use of the term ‘leukemia’ in the names of gammaretroviruses already hints at the most common condition associated with infection. Indeed, malignancies, such as leukemia, lymphoma, and sarcoma, prevail in animals, but immunosuppression and neurological conditions are also seen [1]. The most recent novel primate gammaretrovirus is GALV, whereby infection is either asymptomatic or is associated with acute leukemia and lymphoma [148,265,266]. Recency of the virus is suggested by the lack of endogenous GALV counterparts in the gibbon genome [265,267]. For related KORV, which is a relatively young pathogen of koalas, germline proviruses are already present [268,269]. It brings us to the long-term consequences of retrovirus infections. The tendency to target the early embryo leads to germline integrations that can affect evolution at the species level. Random integrations can activate or inactivate genes, while chromosomal rearrangements can be induced by recombination between different proviruses. Subsequent activation of a provirus can result in changes in protein expression and thus in cellular biology. For the most recently active human ERV family, HERV-K (HML-2), insertional polymorphisms were indeed found to create genetic variation, while specific HML-2 expression profiles are associated with various diseases [270,271,272]. In addition, novel retroviruses might develop through recombination with ERV sequences. Throughout retroviral evolution, env-snatching is common in beta- and gamma-retroviruses, whereby preferentially gamma-type envs are targeted, resulting in type D retroviruses, but also in gamma–gamma recombinants [32,71,273,274,275]. In humans, a gamma-*env* could potentially recombine with HERV-K sequences, of which intact LTRs and genes are encoded in our genome [276,277]. The sarcoma viruses listed by ICTV indicate that gammaretroviruses also recombine with non-ERV host genes. Although such recombinants will need a helper virus for replication, the capacity to shuttle host genes between cells would increase their oncogenic competence.

In the short term, a productive gammaretrovirus infection could lead to significant morbidity and mortality with the possibility of a new virus emerging, which could lead to an epidemic. Should the retrovirus reach the human germline, it is likely to influence the evolution of the species.

## 3. Discussion

There is no evidence that ancestral humans were ever infected by a gammaretrovirus in the past 30 million years, nor that such a virus is present in the population today. Our cousins, the apes, were apparently also spared, except for an encounter with PtERV1 around 3–4 mya [85]. However, gammaretrovirus infections were common in the OWM lineage, with multiple inducible ERVs testifying to the significant virus burden in the recent past. Other retroviruses have had less trouble spreading between OW primates and us, as exemplified by the recent HIV pandemic, and the past and ongoing infections with primate foamy- and deltaretroviruses. For HIV and HTLV, hunting and butchering non-human primates has been proposed as the most likely transmission route, linking diet to infection risk [192,198,199,278]. For SFV, any exposure to non-human primates will possess risk [201,278,279]. The latest primate gammaretrovirus victims are apparently gibbons kept in research facilities. The failure to detect GALV in zoo gibbons, together with a lack of germline integrations, suggests a recent acquisition. Human activity was suspected of being causal here, as blood and tissue from malaria, kuru, and dengue patients, as well as rodent material, were inoculated in the 1960s into gibbons in US medical research facilities in Thailand and in the USA [149,153]. However, a cross-species transmission from a wild animal could likewise be responsible, especially since the Thai institute kept a free-ranging colony of indigenous gibbons. GALV-like replication-competent proviruses can be found in the rodent species *Melomys* and *Mastomys*, although virus isolation from six Australian *Melomys burtoni* blood samples and tissues was unsuccessful [50,145,280]. Since GALV infection has not been reported in gibbons since 1978, and the number of cases was limited anyway, the risk of human infection is estimated to be low [149]. Co-housing of monkeys in research facilities or elsewhere is an effective way of spreading primate retroviruses, as evidenced by SIV transmission from sooty mangabeys to macaques, SRV transmission between macaques, and GALV transmission to a NWM [49,50,51,281,282,283]. Despite the vulnerability of captive non-human primates to retroviruses, human caretakers have not yet found to be infected, except for SFV [201,279,284]. Type D SRVs have historically circulated in captive macaques, but improved diagnostics, selective breeding, and vaccination have nowadays decreased their prevalence significantly [256,285,286]. Research groups have recently reported that, for unexplained reasons, they were unable to infect macaques with SRV, and also that the virus is no longer capable of transmitting between cage mates [287,288]. Should macaques have become less susceptible to SRV infection, then the risk of SRV spreading to humans would decrease concomitantly, although monkeys in the wild may still carry the virus. Further retroviruses with a gamma-type *env* mainly exist in primate DNA as latent but inducible proviruses. Nevertheless, expression in cell culture of, for instance, BaEV, MAC-1, or SERV, requires chemical activation, cocultivation, and/or long-term culture, suggesting that casual contact with monkeys will not be sufficient for transmission.

Interaction with live animals could, theoretically, lead to FeLV transmission from domestic cats, as around 600 million cats around the globe live near humans [289]. FeLV infection is limited to domestic cats and closely related *Felis* species, suggestive of a relatively recent origin [290]. Prevalence of progressive infections varies, whereby the virus is not contained by the immune system but is shed in saliva, for instance. It ranges between 0 and 21% in domestic cats and rises to 28.4% in European wildcats (*Felis sylvestris*) [257,291,292]. Nonetheless, despite the high prevalence in cats and the regular exposure of humans to them, no human FeLV infection has been recorded so far [155,157]. Similarly to the situation with monkey ERVs, the inducible RD-114 provirus is hardly expressed in healthy cats and would therefore not be easily transmitted through direct contact [162]. Exposure to koalas constitutes a risk of contracting KORV, especially since KORV-A has a prevalence of 100% in koala populations, while non-A strain prevalence ranges between 10.3 and 93.9% [293,294,295]. Yet again, human infections have not been reported. As for the avian gammaretrovirus group, it has been suggested that REV was introduced into experimental birds through the inoculation of contaminated *Plasmodium lophurae* stocks in the 1930s [27]. Nowadays, REV spreads in wild and domestic birds as a hitchhiker in the fowlpox genome and in live attenuated fowlpox vaccines, respectively. As avipoxviruses do not infect mammals, human infection through bird exposure is unlikely. However, it would be interesting to analyze human herpesvirus genomes for retroviral integrations, as, for instance, the virulence of Epstein–Barr virus and the specific type of malignancy it is associated with varies globally, which has been attributed to strain variation [296]. A summary of outstanding research questions related to primate gammaretrovirus infections is given in Table 4.

Modern lifestyle, in the form of medical developments, such as xenotransplantation and vector therapy, is a novel risk factor to contract murine or porcine gammaretroviruses, as millennia of close contact with mice and pigs did not result in a human MLV or PERV infection. Fortunately, PERV does not appear to be expressed or transmitted from transplanted pig organs, and PERV-free pigs are available now. Treatment with RRVs is limited at present, not only because people are aware of the risks, but mainly because the therapy failed to meet expectations [297,298]. Still, an analysis of blood samples of clinical trial participants administered a gammaretroviral RRV gives us a glimpse of what could be the early consequences should humans become infected. Transient quantifiable viral DNA levels were seen in blood from a subset of patients, indicative of viral spread but also of systemic control [250]. An autopsy performed on one of the recipients showed low viral DNA levels in spleen, lymph node, liver, and bone marrow, providing further evidence of virus replication and spread [250].

Summarizing all of the above studies, what can humans expect from the gammaretrovirus family in the future? Despite all possible threats from wild and domestic animals, the invention of medical treatments such as vaccination, xenotransplantation, and RRV therapy, and despite the apparently effortless in vitro infection of human cells by most retroviruses with gamma-type *env* genes, a human infection has not been documented yet. Undoubtedly, the past issues with contaminating ‘rumor’ viruses and with HERVs discouraged further human testing. In addition, diagnostic assays may be hindered by low DNA or antibody levels due to suboptimal replication, fast immune control, or the absence of antibodies when infected early in life. In cats, four types of FeLV infection status are recognized, depending on the effectiveness of the immune response, namely, progressively infected, regressively infected, focally infected, and abortively infected [257]. For the latter two types, which are more common than progressive infection, detecting viral DNA can be challenging, as viral DNA is only locally present or is no longer detectable, indicative of virus eradication [257]. Gibbons infected by GALV either develop a persistent antibody response with very low or no measurable viral DNA levels, or, due to the absence of an antibody response, become highly viremic [148,299]. Relying on a single diagnostic test is therefore not enough. To define a human infection, a virus should be characterized; the presence of only antibodies will not suffice. However, antibodies could be all that remains of an infection. Until now, human innate and probably adaptive immune responses have been important factors in combating gammaretrovirus infections. Individuals treated with an MLV RRV suppressed viral replication within weeks, presumably through the generation of neutralizing antibodies [250]. Overall, adequate immune responses may be the most important factor in avoiding gammaretrovirus infection, since humans have certainly met the viruses when hunting monkeys, conducting research on animals, caring for pets, livestock, and poultry, and, in the last century, through medical procedures involving non-human material or replicating viruses. Alternatively, immune responses may have only inhibited large-scale replication, disabling further adaptation of the virus, thus reducing it to a dead-end infection. Since gammaretrovirus infection is commonly associated with leukemia or lymphoma, cancer statistics could be used to investigate potential outbreaks. Current data do not show any remarkable increases in leukemia and lymphoma cases, although, for instance, incidence rates for lymphoma subtypes vary widely between countries and over the years [300,301,302]. So, given that all known gammaretroviruses, including betaretroviruses with gamma-type *env* genes, have apparently not infected humans in the past, and are unlikely to do so in the near future, what would a viral threat from that direction look like? Appreciating the molecular divergence of the viruses listed above, with the fast generation of distinct strains and the recombinant viruses often observed, a novel recombinant virus seems the most plausible prediction. Recombination with ERV sequences is not uncommon and can lead to a virus with novel characteristics. An alternative could be a surprise virus from an unexpected source. For instance, dogs, our long-time companions, harbor replication-competent gamma-ERVs, which are highly expressed in dogs with leukemia and lymphoma [209]. Should such a virus end up in an individual with a genetic flaw, leading to an inadequate immune response, but being capable of living a long and fruitful life, a new human pathogen may be born. But for now, the conclusion should be that we humans have indeed evaded gammaretrovirus infections for a very long time, but the situation may change in the future, whereby not an ICTV-listed virus, but a novel ERV-recombinant or a virus from an unexpected source would be the most likely candidate, unless we keep treating humans with RRVs.

## Figures and Tables

**Table 1:** Gamma- and betaretroviruses included in the review.

Retrovirus Group	Virus Name *	Abbreviation *	Host Species
ICTV exemplar gammaretroviruses	reticuloendotheliosis virus	REV	bird
	Trager duck spleen necrosis virus	SNV	bird
	chick syncytial virus	CSV	bird
	murine leukemia virus	MLV	mouse
	feline leukemia virus	FeLV	cat
	gibbon ape leukemia virus	GALV	gibbon
	koala retrovirus	KoRV	koala
	porcine type-C oncovirus	PCOV/PERV	pig
ICTV exemplar gammaretroviruses needing a helper virus	Finkel–Biskis–Jinkins murine sarcoma virus	-	mouse
	Harvey murine sarcoma virus	-	mouse
	Kirsten murine sarcoma virus	-	mouse
	Moloney murine sarcoma virus	-	mouse
	Hardy–Zuckerman feline sarcoma virus	-	cat
	Snyder–Theilen feline sarcoma virus	-	cat
	woolly monkey sarcoma virus	WMSV	woolly monkey
ICTV exemplar betaretroviruses with a gamma-type *env* gene	Po-1-Lu	Po-1-Lu	langur monkey
	Mason–Pfizer monkey virus	MPMV/SRV3	rhesus monkey
	squirrel monkey retrovirus	SMRV	squirrel monkey
Non-ICTV gammaretroviruses	baboon endogenous virus	BaEV	baboon
	MAC-1	MAC-1	rhesus monkey
	CPC-1	CPC-1	colobus monkey
Non-ICTV betaretroviruses with a gamma-type *env* gene	simian endogenous retrovirus	SERV	African green monkey
	RD-114	RD-114	cat

* Common name/abbreviation, which is not the official ICTV name/abbreviation since 2023.

**Table 2:** Retroviruses with a gamma-type *env* gene were detected in human samples and cell lines.

Gammaretrovirus	Source	Year [Reference]
RD-114	Passaging of human tissue in kittens	1972 [94]
HL23V (GALV variant)	Lab contamination	1975 [98,99]
BaEV + MLV variants	Lab contamination	1976/1977 [105,106]
SMRV	Lab contamination	1988 [124]
MSRV	HERV-W	1991 [110]
GALV	Lab contamination	1993 [120]
XMRV	Lab contamination	2006 [117]
MLV/GALV	Lab contamination screening	2008 [122]
Synthetic MLV variant	Lab contamination	2009 [123]
SMRV	Lab contamination screening	2010 [125]
MLV	Lab contamination screening	2015 [126]

**Table 3:** Possible sources of gammaretrovirus transmission to humans with recommendations to decrease risks.

Gammaretrovirus ^1^	Possible Infection Source	Recommendation
Avian gammaretroviruses (REV, SNV, CSV)	Poultry products	Screen flocks
GALV	Contact with gibbons Clinical use of RRVs	Protective measures ^2^ Limit use
MLV	Clinical use of RRVs	Limit use
FeLV	Contact with pet cats	Vaccinate cats ^3^
KORV	Exposure to koalas	Protective measures Vaccinate koalas ^4^
PERV/PCOV	Xenotransplantation	Edit pig genome
SRV1-3	Exposure to primates	Protective measures ^2^
Primate ERVs ^5^ (Po-1-Lu, SMRV, BaEV, MAC-1, CPC-1, SERV)	Exposure to primates Cell line products	Protective measures Perform quality checks
Cat ERV RD-114	Cell line products	Edit cell line genome
‘Sarcoma’ retroviruses	None	None

^1^ The color of the row indicates the level of endogenization, with white implying no endogenization in the original animal source, light gray depicting viruses with both endogenous and exogenous variants, and dark gray representing endogenous proviruses that are activated only under specific conditions. ^2^ GALV infection in gibbons has not been reported since the 1970s. SRV prevalence in captive monkeys has declined significantly after 1990 [256]. ^3^ Cat vaccines are available in most countries and are strongly advised [257]. ^4^ No vaccine has been marketed yet, but a therapeutic vaccine proved to be effective [258]. ^5^ Xenotransplantation of baboon tissue has been discontinued.

**Table 4:** Outstanding research questions and directions for future research.

Human gammaretrovirus infection	Local leukemia and lymphoma incidence variation could be used to guide testing of human samples. Which assays should be used for testing? Targeted screening of sequence datasets could be useful.
GALV	What has happened to GALV since 1978? Are gibbons living in the wild infected by GALV?
SRV	What is the origin of the SRV strains? Why can research monkeys no longer be infected with SRV? Can germline SRV proviruses be found in captive monkeys?
Retroviral integration	Are retroviruses integrated in human herpesvirus genomes?

## Data Availability

No new data were created or analyzed in this study. Data sharing is not applicable to this article.

## Abbreviations

The following abbreviations are used in this manuscript:

APOBEC3	Apolipoprotein B mRNA editing enzyme, catalytic polypeptide 3
DNA	Deoxyribonucleic acid
HUSH	Human silencing hub
PBMC	Peripheral blood mononuclear cells
PCR	Polymerase chain reaction
RNA	Ribonucleic acid
SAMHD1	SAM domain and HD domain-containing protein 1
SERINC5	Serine incorporator 5
TRIM5α	Tripartite motif-containing protein 5α
ZAP	Zinc finger antiviral protein
